# Supplementary material for: Natural dynamics and watershed approach incorporation in urban water management: A scoping review
Source: PLoS One. 2024 Aug 30;19(8):e0309239. doi: 10.1371/journal.pone.0309239 (PMC11364295; doi:10.1371/journal.pone.0309239)
Supplement: S2 File — (DOCX) [file pone.0309239.s002.docx]

| **S2. Table**. Descriptive summary of the documents included in this scoping review. An "**X**" indicates the information obtained from each document related to the categories definedin the research question (drivers, actions, challenges and results). Some examples of the information obtained from each document are provided in parentheses. | | | | | | | | | | |
| --- | --- | --- | --- | --- | --- | --- | --- | --- | --- | --- |
| **#** | **TITLE** | **AUTHORS** | **YEAR** | **TYPE OF DOCUMENT** | **COUNTRY** | **CITY** | **IDENTIFIED INFORMATION** | | | |
|  |  |  |  |  |  |  | **DRIVERS** | **ACTIONS** | **CHALLENGES** | **RESULTS** |
| **1** | Green infrastructure for sustainable urban water management: Practices of five forerunner cities. | Liu L, Jensen MB. | 2018 | Academic article | China | Tianjin | **X** (increasing water supply and green-blue spaces maintenance) | **X** (green areas irrigated by mainly reclaimed water, rainwater harvesting, storm-water collection, constructed wetlands for cleansing collected stormwater runoff and small water landscapes) | **X**  (lack of construction and maintenance experiences, technical, operational and financial challenges) |  |
|  |  |  |  |  | Singapore | Singapore | **X**  (increasing water supply, water security and flood control) | **X**  (water alternative sources with rainwater collection, reclaimed wastewater, desalinated water, and imported water; green infrastructure such as eco-districts and green-buildings programs) | **X**  (technical, spacial, financial and time challenges for implementing the actions) | **X**  (increase of water supply alternatives: 60% water supply from non-conventional sources of which 30% is reclaimed, 20% is rainwater, and 10% is desalinated water) |
|  |  |  |  |  | Australia | Melbourne | **X**  (increasing water supply, environment protection particularly of rivers and flood control) | **X** (pilot projects for rainwater collection for irrigation, runoff cleanup for stream protection, and development of watershed management strategies) | **X**  (institutional challenges, barriers for multidisciplinary partnerships, negative opinion about unconventional water, conflicts with existing infrastructure, and maintenance challenges) |  |
|  |  |  |  |  | Germany | Berlin | **X** (increasing water supply, flood control, social pressure for bathing and recreation in surface waters and financial support from the national government) | **X**  (green infrastucture, pilot projects for eco-solutions, norms with criteria, indicators and guidelines for urban planning and design) | **X**  (coordination problems, stakeholders opposition and limitation of the existing legislations) | **X**  (100% water supply from sustainable sources; ecosystem services recovery) |
|  |  |  |  |  | United States of America | Philadelphia | **X**  (environmental protection; recreation and flood control) | **X**  (green infrastucture) | **X**  (logistics, coordination, social financial and operational challenges for implementing the actions and lack of information to assess the level of local water balance due to dependence on regional centralized supply) |  |
| **2** | Centralized water reuse system with multiple applications in urban areas: Lessons from China’s experience. | Chen Z, Wu Q, Wu G, Hu HY. | 2017 | Academic article | China | Tianjin | **X** (increasing water supply with alternative water sources and strong government support) | **X**  (water reuse) |  | **X**  (increased water resource availability based on water reuse) |
| **3** | Implementation of a specific urban water management - Sponge City | Nguyen TT, Ngo HH, Guo W, Wang XC, Ren N, Li G, et al. | 2019 | Academic article | China | Shanghai | **X**  (national government political and financial support and selection of thirty urban areas as pilot Sponge Cities and urban water-related problems) | **X** (green roofs, green spaces, artificial rainwater wetlands, infiltration ponds and biological retention facilities and water-permeable paving) | **X**  (technical, operational and maintenance challenges) |  |
|  |  |  |  |  |  | Chongqing |  |  |  |  |
|  |  |  |  |  |  | Wuhan |  |  |  |  |
|  |  |  |  |  |  | Nanning |  |  |  |  |
|  |  |  |  |  |  | Ningbo |  |  |  |  |
|  |  |  |  |  |  | Shenzhen |  |  |  |  |
| **4** | Urban water security assessment in the context of sustainability and urban water management transitions: An empirical study in Shanghai. | Zhu D, Chang YJ. | 2020 | Academic article | China | Shanghai | **X**  (water quality improvement) | **X**  (regulations and policies for water pollution prevention and control and actions such as wastewater treatment and water quality monitoring) |  | **X**  (water quality improvement illustrated based on river quality data that have shown slight enhancement) |
|  | Regenerating Sponge City to Sponge Watershed through an Innovative Framework for Urban Water Resilience. | Wang J, Xue F, Jing R, Lu Q, Huang Y, Sun X, et al. | 2021 | Academic article | China | Shanghai | **X** (national government political and financial drivers based on the strategy for the Sponge City construction; 60–90 million USD per year for three years to support pilot cities) | **X**  (local norms that establish a strict control of composite runoff coefficient in newly built areas) | **X**  (institutional coordination, financial efficiency and sustainability of projects in all cities; in the case of Chongqing the landform of mountain terrain increases the difficulty of runoff flow, velocity control and water quality control) | **X**  (remarkable results in water treatment and water ecology modules) |
| **5** |  |  |  |  |  | Chongqing |  |  |  |  |
|  |  |  |  |  |  | Wuhan |  |  |  | **X** (good performance in water resilience, water resource and water management) |
|  |  |  |  |  |  | Nanning |  |  |  | **X**  (rainwater utilization and ecological water replenishment progress based on higher mean annual precipitation) |
|  |  |  |  |  |  | Ningbo |  | **X**  (local norms that establish that the comprehensive runoff coefficient of the main concentrated land should not be greater than 0.6) |  | **X**  (runoff control and good performance in water resilience, water resources, water treatment, and water landscape modules). |
|  |  |  |  |  |  | Shenzhen |  | **X**  (optimization of ecological green corridors, urban water system planning and openings along rivers within the city) |  | **X**  (contribution to runoff control and outstanding leverages concentrated in the modules of water resilience and water management) |
|  |  |  |  |  | Singapore | Singapore |  | **X**  (urban rainwater collection and drainage network throughout the island) |  | **X**  (waterlogging and flooding prevention even with heavy storms) |
| **6** | Addressing Challenges of Urban Water Management in Chinese Sponge Cities via Nature-Based Solutions. | Qi Y, Chan FKS, Thorne C, O’Donnell E, Quagliolo C, Comino E, et al. | 2020 | Academic article | China | Chongqing | **X**  (flood control and national government political and financial drivers with the Sponge City Program) | **X** (stormwater management and blue-green infrastructure) | **X**  (financial barriers and geographical challenges due to contrasting geographical areas; lack of a basin level vision and climate change risks considerations in projects) |  |
|  |  |  |  |  |  | Wuhan |  |  |  |  |
|  |  |  |  |  |  | Ningbo |  |  |  |  |
|  |  |  |  |  |  | Shenzhen |  |  |  |  |
| **7** | Assessment of water resource security in Chongqing City of China: What has been done and what remains to be done? | Zhang JY, Wang LC. | 2015 | Academic article | China | Chongqing | **X**  (water security based on water availability and water quality) | **X**  (ecosystem conservation and restauration such as the ecological management of the Three Gorges Reservoir) |  | **X** (water security exhibits an improving trend related to the increase in the percentage of forest coverage and the ecological management of the Three Gorges Reservoir) |
| **8** | Regional Water Policy in China – Problems and Approaches in the Taihu und Wuhan Regions. | Dai L, Qin T. | 2019 | Book chapter | China | Wuhan | **X**  (increasing water supply and quality; political and financial drivers promoted by municipal and national government) | **X**  (waterlogging and nonpointsource pollution control; rainwater collection and reuse) |  |  |
| **9** | Building Climate Resilience and Water Security in Cities: Lessons from the sponge city of Wuhan, China. | Oates L, Dai L, Sudmant A, Gouldson A. | 2020 | Academic article | China | Wuhan | **X**  (political drivers for sponge city projects such as financial and technical support from the national government; floods control; water infiltration) | **X**  (389 sponge city projects covering 38.5 km2, such as urban gardens, parks and green spaces for water infiltration, artificial lakes and water channels) |  | **X**  (increased water infiltration, improved local air quality, ecosystem conservation benefits, social benefits, and increased land value; but lack of information of sponge city impacts at the basin level) |
| **10** | International Perceptions of Urban Blue-Green Infrastructure: A Comparison across Four Cities. | O’Donnell EC, Netusil NR, Chan FKS, Dolman NJ, Gosling SN. | 2021 | Academic article | China | Ningbo | **X**  (increasing water supply with rainwater absorption, water storage, purification and reuse; and flood control; political drivers with Sponge City Program) | **X**  (urban planning incorporating low impact development for improving the capacity of water absorption) |  |  |
|  |  |  |  |  | Netherlands | Rotterdam | **X**  (flood control and increasing the resilience to climate change future impacts) | **X**  (norms and policies for coordinating urban water management, spatial planning and climate change adaptation actions; specific actions such as green roofs and rooftop parks) |  |  |
|  |  |  |  |  | United States of America | Portland, Oregon | **X**  (flood control, water quality, and enhancing fish habitat; economic drivers such as benefits and costs of blue and green infrastructure; social drivers such as nonprofits and citizen advocacy) | **X** (norms and policies such as the “Grey to Green initiative” for turning grey to green and blue infrastructure; specific actions as ecoroofs and green streets, ecosystem restoration of urban streams and native vegetation) |  |  |
| **11** | Aligning ancient and modern approaches to sustainable urban water management in China: Ningbo as a “Blue-Green City” in the “Sponge City” campaign. | Tang YT, Chan FKS, O’Donnell EC, Griffiths J, Lau L, Higgitt DL, et al. | 2018 | Academic article | China | Ningbo | **X** (increasing water supply and water quality, reducing water demand, flood control and political incentives from the province and national level) | **X** (green and blue insfrastructure such as the “Eco-corridor” with features including woodlands, rain-gardens, cascades of wetlands, meandering streams and open water areas) | **X**  (urbanization conversion into gray infrastructure of several blue and green areas in the city increase the challenges to retrofit those changes; lack of social engagement) |  |
| **12** | Good Practices in Urban Water Management: Decoding Good Practices for a Successful Future. | Chiplunkar A, Seetharam K, Kheong Tan C, editors. | 2012 | Book | China | Shenzhen | **X** (droughts and floods control, legal and political drivers at the national and city level, institutional drivers from the combination of all water-related government functions into one governmental agency) | **X**  (wastewater treatment, integrated water management of water supply and drainage) | **X** (challenges for water quality improvement in rivers due to the city’s natural and geographical conditions, such as river channels that are short and have limited self-purification capacity) | **X**  (increase in wastewater treatment rate and total annual water supply) |
|  |  |  |  |  | Singapore | Singapore | **X**  (water scarcity and vulnerability and political commitment for water security) | **X**  (supply management including catchment management, wastewater and stormwater management and demand management including water pricing and education programs) | **X** (social perceptions related to water reuse) | **X**  (improvements in water availability and quality, for instance, all the population has access to high-quality piped water on a 24-hour basis daily) |
| **13** | Sponge City Policy and Sustainable City Development: The Case of Shenzhen. | Wang Y, Jiang Z, Zhang L. | 2022 | Academic article | China | Shenzhen | **X**  (political drivers at the national level with the Sponge City Program) | **X**  (1,361 projects of green infrastructure and Low Impact Development including the Baishida Elementary School project with a permeable playground to solve water accumulation and the Vanke Cloud City Project) |  | **X**  (increase of the water infiltration areas with newly built-up sponge city area that is 276 km2 accounting for 28.3% of the urban area; 220 historical waterlogging points eliminated; however the monitoring data of the sponge city is insufficient and lacks systematic rainwater management database) |
| **14** | Role of Sponge City Development in China’s battle against urban water pollution: Insights from a transjurisdictional water quality management study. | Xiong J, Zheng Y, Zhang J, Xu P, Lu H, Quan F, et al. | 2021 | Academic article | China | Shenzhen | **X**  (water pollution, political and economical incentives bases on national government support for Sponge City pilots) | **X**  (green infrastructure such as green roofs, bioretention cells, porous pavements and constructed wetlands) |  | **X**  (potential to reduce water pollution but knowledge gaps remain in understanding the level of impact) |
| **15** | Assessing Urban Water Management Sustainability of a Megacity: Case Study of Seoul, South Korea. | Kim H, Son J, Lee S, Koop S. | 2018 | Academic article | South Korea | Seoul | **X**  (population growth, water-related challenges, and climate change risks) | **X**  (wastewater treatment plants) | **X**  (economic barriers and lack of general public concern about water-related challenges) |  |
| **16** | History of Water Sensitive Urban Design/Low Impact Development Adoption in Australia and Internationally. | Radcliffe JC. | 2019 | Book chapter | South Korea | Seoul | **X**  (flood control, increase water infiltration and runoff control) | **X** (actions for rainfall collection such as a Rainfall-Storage-Drain modeling system in high density and impermeable areas to control runoff) |  |  |
|  |  |  |  |  | United States of America | New York | **X**  (national regulations such as te US Federal Clean Water Act) | **X** (norms and policies for coordinating urban water management such as the Protection Strategic Plan 2011-14 and the National Municipal Stormwater Alliance) |  |  |
| **17** | Recent Progress in Low-Impact Development in South Korea: Water-Management Policies, Challenges and Opportunities. | Shafique M, Kim R. | 2018 | Academic article | South Korea | Seoul | **X**  (normative drivers such as water regulation plans including LID practices) | **X**  (plans and regulations such as a comprehensive plan for the healthy water-cycle city; and specific actions such as green infrastructure, green and blue roofs and rainwater management) | **X**  (lack of collaboration, knowledge and operation capacities in institutions and market barriers) | **X**  (total rainfall runoff volume reduction value of stormwater management) |
| **18** | Total water management and water sensitive cities. | Gamage DR. | 2014 | Conference Paper | Singapore | Singapore | **X**  (droughts problems, flood control, and economic incentives such as rising energy costs for operation and maintenance of the water system) | **X**  (actions to increase the collection and reuse of rainwater, for instance, it has increased its rainwater catchment area from a half to two-thirds of its total land surface) |  |  |
|  |  |  |  |  | Australia | Melbourne | **X**  (increasing water security, livability and recreational use of green and blue areas, and address higher costs of water management) |  |  |  |
|  |  |  |  |  | Netherlands | Rotterdam | **X**  (climate change resilience connected to the need of protecting its port, and increase liveability of the city) | **X**  (actions such as a water square which provides both water storage and leisure areas) |  |  |
|  |  |  |  |  | United States of America | Portland, Oregon | **X**  (increasing water quality and urban liveability) | **X**  (policies and programs to promote stormwater solutions such as green roofs and disconnected downspouts) |  |  |
| **19** | Institutional capacity and policy options for integrated urban water management: a Singapore case study. | Chen DC, Maksimovic C, Voulvoulis N. | 2011 | Academic article | Singapore | Singapore | **X** (water security, expand alternative water sources, institutional and political drivers) | **X**  (plans and policies for integrating land use planning and water resource management; specific actions such as storm water and wastewater management, treatment and reuse) |  |  |
| **20** | Tale of Two Cities: How Nature-Based Solutions Help Create Adaptive and Resilient Urban Water Management Practices in Singapore and Lisbon. | Cui M, Ferreira F, Fung TK, Matos JS. | 2021 | Academic article | Singapore | Singapore | **X**  (political and institutional drivers such as multi-governmental collaboration, and economic investment) | **X**  (actions to collect, store and protect rainwater and to collect and treat wastewater) |  |  |
| **21** | Integrated Urban Water Management and Water Security: A Comparison of Singapore and Hong Kong. | Jensen O, Nair S. | 2019 | Academic article | Singapore | Singapore | **X** (water security based on geopolitical incentives for avoiding water dependency from a foreign source; increase water supply; political and institutional incentives based on political will and coordination between water and urban planning agencies) | **X**  (actions for increasing alternative water sources such as water reuse and desalination) |  |  |
| **22** | Closing the urban water loop: lessons from Singapore and Windhoek. | Lafforgue M, Lenouvel V. | 2015 | Academic article | Singapore | Singapore | **X**  (water security based on geopolitical incentives for avoiding water dependency from a foreign source) | **X**  (actions for reducing the water demand -incentive-based billing, communication campaigns and water-saving devices- and increasing water availability -rainwater harvesting, wastewater recycling and seawater desalination) | **X**  (increased energy consumption for water management and treatment) | **X**  (increased water availability; one quarter of the water used in Singapore is derived from recycled wastewater) |
| **23** | Applications of Nature-Based Solutions in Urban Water Management in Singapore, Thailand and Vietnam: A Review. | Linh NS, Ahmed F, Loc HH. | 2022 | Book chapter | Singapore | Singapore | **X**  (water security, institutional and regulatory incentives) | **X**  (nature based solutions converting the grey infrastructure to green infrastructure such as Rain Gardens in Schools) |  |  |
| **24** | Water quality management in Singapore: the role of institutions, laws and regulations. | Tortajada C, Joshi YK. | 2014 | Academic article | Singapore | Singapore | **X**  (institutional drivers such as the institutional integration for water management and normative drivers with a strong enforcement of laws related to water) | **X**  (plans and regulations such as the Green Plan for Environmental Protection and Improvement) |  |  |
| **25** | The enabling institutional context for integrated water management: Lessons from Melbourne. | Ferguson BC, Brown RR, Frantzeskaki N, De Haan FJ, Deletic A. | 2013 | Academic article | Australia | Melbourne | **X**  (drought problems, normative, institutional and social drivers, and economic incentives) | **X**  (policies and norms promoting alternative water sources such as stormwater harvesting technologies) | **X**  (lack of knowledge, capacities and organisational structures needed for the implementation of water management changes) |  |
| **26** | Governance of Integrated Urban Water Management in Melbourne, Australia. | Furlong C, Gan K, De Silva S. | 2016 | Academic article | Australia | Melbourne | **X** (drought problems, public awareness of water issues, political drivers, and economical drivers such as subsidies for water saving) | **X**  (rainwater tanks, stormwater harvesting and wastewater recycling) |  |  |
| **27** | Key concepts for Integrated Urban Water Management infrastructure planning: Lessons from Melbourne. | Furlong C, Brotchie R, Considine R, Finlayson G, Guthrie L. | 2017 | Academic article | Australia | Melbourne | **X**  (institutional and governmental drivers) | **X**  (norms, plans and policies related to water management; and specific actions such as stormwater harvesting projects and wastewater reuse projects) | **X**  (lack of consideration of future uncertainty and climate scenarios and lack of capacities for financial and technical analysis) |  |
| **28** | Infrastructure and Urban Planning Context for Achieving the Visions of Integrated Urban Water Management and Water Sensitive Urban Design: The Case of Melbourne. | Furlong C, Dobbie M, Morison P, Dodson J, Pendergast M. | 2019 | Book chapter | Australia | Melbourne | **X** (legislation and policy drivers) | **X**  (norms, plans and policies related to urban water management) | **X** (lack of clarity and ambiguity in norms, legislation and policies related to implementation actions and who is responsible for maintenance; competing interests and conflicts among stakeholders) |  |
| **29** | The role of business models and transitional pressures in attaining sustainable urban water management. | Marlow DR, Müller NA, Moglia M. | 2017 | Academic article | Australia | Melbourne | **X** (drought problems and population increase; political and social drivers related to the central priority given to water issues) | **X**  (stormwater and rain water harvesting) |  |  |
| **30** | New paradigms in urban water management for conservation and sustainability. | Capodaglio AG, Ghilardi P, Boguniewicz-Zablocka J. | 2016 | Academic article | Germany | Berlin | **X** (geopolitical and historical incentives for water security, social demands for sustainable urban policies, political and financial incentives from the national government) | **X**  (riverbank filtration, green roofs, sustainable rainwater management and water demand management) |  | **X** (rainwater infiltration and groundwater resources increased, reduction of per capita water consumption from 250 to 112 litres/person/day and economic savings) |
| **31** | Transforming water infrastructure by linking water management and urban renewal in Rotterdam. | de Graaf R, van der Brugge R. | 2010 | Academic article | Netherlands | Rotterdam | **X**  (climate change resilience, social and institutional drivers) | **X**  (policy plans and initiatives such as the Rotterdam Water City 2035 and specific actions such as green roofs) |  |  |
| **32** | The role of science-policy interface in sustainable urban water transitions: Lessons from Rotterdam. | Dunn G, Brown RR, Bos JJ, Bakker K. | 2017 | Academic article | Netherlands | Rotterdam | **X**  (flood control, climate change resilience, liveability and political incentives) | **X**  (policy plans such as Rotterdam Water Plan and initiatives as the Rotterdam Water City 2035) |  |  |
| **33** | Linking water policy innovation and urban renewal: the case of Rotterdam, The Netherlands. | van der Brugge R, de Graaf R. | 2010 | Academic article | Netherlands | Rotterdam | **X**  (flood control, droughts and climate change resilience; change agents at the local level from governmental and non governmental sectors) | **X**  (policy plans such as water management plans and initiatives such as Rotterdam Water City 2035; and specific actions such as blue areas recovery and storm water reuse and treatment) | **X** (lack of institutional mechanisms for wider implementation of actions regarding investments, operation and maintenance; key stakeholders which are not involved) | **X**  (increase in water quality, for instance, sewer emissions into the river were successfully reduced) |
| **34** | Adapting to Climate Change in Urban Water Management: Flood Management in the Rotterdam–Rijnmond Area. | van Vliet M, Aerts JCJH. | 2015 | Book chapter | Netherlands | Rotterdam | **X**  (flood control) | **X** (policies and regulations with topics such as flood zoning and building codes; and specific actions such as green roofs) | **X**  (laws and regulations which are not always clear, law enforcing challenges and social opposition to some policies) |  |
| **35** | New Planning Activities in Vienna’s Water-Management. | Hozang B. | 2018 | Book chapter | Austria | Vienna | **X**  (recovery of ecosystems and water quality, liveability and recreational activities in blue and green spaces) | **X**  (lake ‘Alte Donau’ and its surrounding green spaces recovery and conservation actions based on the Masterplan Alte Donau) |  | **X**  (successful water quality safeguarding and ecosystem restauration progress) |
| **36** | Aquatic habitats in Vienna (Austria) - integrating ecology and urban water management. | Janauer GA. | 2005 | Academic article | Austria | Vienna | **X**  (social opinion, political priorities, recreational activities in blue and green spaces) | **X**  (ecosystem rehabilitation actions) |  | **X**  (recreational use of green and blue areas restaured re-established) |
| **37** | Improving health in cities through systems approaches for urban water management. | Rietveld LC, Siri JG, Chakravarty I, Arsénio AM, Biswas R, Chatterjee A. | 2016 | Academic article | Austria | Vienna | **X**  (flood control, recreational activities in blue and green spaces, interdisciplinary teams supporting the actions related to engineering, architecture, landscape, ecology, limnology, and water issues) | **X**  (protection of green areas for recreation and ecosystem conservation, for instance, the creation of a second parallel river to act as a flood channel, separated from the main channel, with natural filtration systems) |  |  |
| **38** | A breakthrough in urban rain-harvesting schemes through planning for urban greening: Case studies from Stockholm and Barcelona. | Suleiman L, Olofsson B, Saurí D, Palau-Rof L. | 2020 | Academic article | Sweden | Stockholm | **X**  (air pollution problem and political support) | **X** (specific actions of rain-harvesting such as the "Environmental Rehabilitation of Hornsgatan") | **X**  (lack of technical knowledge, experience and sensitivity to implement the project, such as the resistance from civil engineers to the project, and cost barriers) | **X**  (increase of quantity and quality in the water infiltrated and social benefits such as safety and improved air quality, and replicability of the project) |
| **39** | Sustainable Flood Risk and Stormwater Management in Blue‐Green Cities; an Interdisciplinary Case Study in Portland, Oregon. | O’Donnell EC, Thorne CR, Yeakley JA, Chan FKS. | 2020 | Academic article | United States of America | Portland, Oregon | **X** (political and normative drivers based on federal regulations, flood control, climate change resilience and liveability) | **X**  (plans and projects such as the “Grey to Green” initiative, and specific actions such as the implementation of green infrastructure to manage stormwater, 2,000 street bioswales, more than 600 ecoroofs and tens of thousands of street trees, restoration of urban watercourses, and improvements to water quality and aquatic environments) |  |  |
| **40** | Clean Waters, Clean City: Sustainable Storm Water Management in Philadelphia. | Mandarano L. | 2011 | Book chapter | United States of America | Philadelphia | **X**  (increasing water quality and water supply, and facing health issues related to water problems) | **X**  (green infrastructure, LID techniques, natural areas conservation and storm water regulations for urban developers) |  |  |
| **41** | Integrated Urban Water Management. | Bahri A. | 2012 | Technical report | United States of America | New York | **X**  (economical, institutional and normative incentives based on national regulations) | **X**  (regulatory programs and specific actons such as treatment systems, stormwater and wastewater treatment infrastructure and watershed protection actions) |  |  |
