# Supplementary material for: Natural dynamics and watershed approach incorporation in urban water management: A scoping review
Source: PLoS One. 2024 Aug 30;19(8):e0309239. doi: 10.1371/journal.pone.0309239 (PMC11364295; doi:10.1371/journal.pone.0309239)
Supplement: S3 File — (DOCX) [file pone.0309239.s003.docx]

| **S3. Table. Studies identified in the literature search** | | | |
| --- | --- | --- | --- |
| **#** | **Title** | **Authors** | **Year** |
| **1** | Water shortage management system and challenges in water scarcity area of Haramaya watershed, Eastern Ethiopia | Tasisa Temesgen | **2023** |
| **2** | Assessment and Actions to Support Integrated Water Resources Management of Seville (Spain) | Algaba, Maria Hernandez-Pacheco; Huyghe, Wim; van Leeuwen, Kees; Koop, Stef; Eisenreich, Steven | **2023** |
| **3** | Calibration of SWMM's hydrological model for the watershed vai e volta stream - Pocos de Caldas, Minas Gerais | Silveira, A; Cavalcanti, AA; de Menezes, FCM; Severino, MF | **2022** |
| **4** | Achieving Urban Water Security in Tokyo | Chattha, M. K., Wei, Z., & Swatuk, L. | **2022** |
| **5** | Denver One Water Plan — Setting the stage for broad utility collaboration | Wiersema, I., Rehring, J., Jula, D., & Bereskie, T. | **2022** |
| **6** | Nature-based solutions as water management measures in Hungary | Balatonyi, László1 – Lengyel, Bálint2 – Berger, Ádám3 | **2022** |
| **7** | Effects of land-cover and watershed protection futures on sustainable groundwater management in a heavily utilized aquifer in Hawai'i (USA) | Bremer, LL; Elshall, AS; Wada, CA; Brewington, L; Delevaux, JMS; El-Kadi, AI; Voss, CI; Burnett, KM | **2021** |
| **8** | Upper Santa Ana River watershed - integrated regional urban water management plan | Lehman, H. | **2021** |
| **7** | Local Government Perspectives on Collaborative Governance: A Comparative Analysis of Iowa's Watershed Management Authorities(sic)(sic)(sic)Palabras claves | Yoder, L; Ward, AS; Spak, S; Dalrymple, KE | **2021** |
| **8** | Delving into the Divisive Waters of River Basin Planning in Bolivia: A Case Study in the Cochabamba Valley | Nilo Lima-Quispe, Cláudia Coleoni, Wilford Rincón, Zulema Gutierrez, Freddy Zubieta, Sergio Nuñez, Jorge Iriarte, Cecilia Saldías, David Purkey, Marisa Escobar and Héctor Angarita | **2021** |
| **9** | Regenerating Sponge City to Sponge Watershed through an Innovative Framework for Urban Water Resilience | Jian Wang, Fei Xue, Ruiying Jing, Qiaohui Lu, Yilong Huang, Xiang Sun and Wenbo Zhu | **2021** |
| **10** | Optimization of wastewater treatment strategies using life cycle assessment from a watershed perspective | Bai, SW; Tu, YN; Sun, HL; Zhang, HQ; Yang, SS; Ren, NQ | **2021** |
| **11** | Best Management Practices for the Transition to a Water-Sensitive City in the South of Portugal | Rodrigues, M; Antunes, C | **2021** |
| **12** | Alternative storm water management scenarios for developing countries in urban contexts | Gogate, NG; Jedhe, Y | **2021** |
| **13** | River Restoration Integrated with Sustainable Urban Water Management for Resilient Cities | Aline Pires Veról, Ianic Bigate Lourenço, João Paulo Rebechi Fraga, Bruna Peres Battemarco, Mylenna Linares Merlo, Paulo Canedo de Magalhães and Marcelo Gomes Miguez | **2020** |
| **14** | Energy use for urban water management by utilities and households in Los Angeles | Erik Porse, Kathryn B Mika, Alvar Escriva-Bou, Eric D Fournier, Kelly T Sanders, Edward Spang, Jennifer Stokes-Draut, Felicia Federico, Mark Gold and Stephanie Pincetl | **2020** |
| **15** | Comparing Complexity in Watershed Governance: The Case of California | Ulibarri, Nicola; Garcia, Nataly Escobedo | **2020** |
| **17** | Integrated ecological modelling for evidence-based determination of water management interventions in urbanized river basins: Case study in the Cuenca River basin (Ecuador) | Jerves-Cobo, R; Benedetti, L; Amerlinck, Y; Lock, K; De Mulder, C; Van Butsel, J; Cisneros, F; Goethals, P; Nopens, I | **2020** |
| **18** | A Socio-Ecological System Analysis of Multilevel Water Governance in Nicaragua | Luis Montenegro and Jochen Hack | **2020** |
| **19** | Evaluation of the water resource plans in Turkey based on sustainable water management principles | Pouya, S; Turkoglu, H | **2020** |
| **20** | Establishment and implementation of green infrastructure practice for healthy watershed management: Challenges and perspectives | Bo-Wei Liu a, Ming-Huang Wang b, Tse-Lun Chen c, Po-Chih Tseng c, Yongjun Sun d, Andrew Chiang e, Pen-Chi Chiang c | **2020** |
| **21** | A breakthrough in urban rain-harvesting schemes through planning for urban greening: Case studies from Stockholm and Barcelona | Lina Suleiman, Bo Olofsson, David Saurí, Laura Palau-Rof | **2020** |
| **22** | Managing socio-ecological systems: who, what and how much? The case of the Banas River, Rajasthan, India | Everard, M | **2020** |
| **23** | Sustainable Surface Water Management and Wastewater Treatment Plant Location: A Case Study of Urmia Lake | Azizifard, A; Arkat, J; Farughi, H | **2020** |
| **24** | Validation of the Three-Step Strategic Approach for improving urban water management and water resource quality improvement | Galvis-Castano, A | **2019** |
| **25** | Sustainable urban water management and integrated development in informal settlements: The contested politics of co-production in Santo Domingo, Dominican Republic | Sletto, B; Tabory, S; Strickler, K | **2019** |
| **26** | Interdisciplinary Collaboration on Green Infrastructure for Urban Watershed Management: An Ohio Case Study | Shawn Dayson Shifflett, Tammy Newcomer-Johnson, Tanner Yess and Scott Jacobs | **2019** |
| **27** | Investing upstream: Watershed protection in Piura, Peru | Abby Lindsay Ostovar | **2019** |
| **28** | Implementation of a specific urban water management - Sponge City | Thu Thuy Nguyen a, Huu Hao Ngo a, Wenshan Guo a, Xiaochang C. Wang b, Nanqi Ren c, Guibai Li c, Jie Ding c, Heng Liang c | **2019** |
| **29** | Urban Water Management Paradigms in Chinese Cities | Meine Pieter van Dijk and Mingshun Zhang | **2019** |
| **30** | The Eco-Compensation Mechanism in Tai Lake Watershed | Dai, LP; Qin, TB | **2019** |
| **31** | Situations, challenges and strategies of urban water management in Beijing under rapid urbanization effect | Liu, W; Chen, WP; Feng, Q; Deo, RC | **2019** |
| **32** | History of Water Sensitive Urban Design/Low Impact Development Adoption in Australia and Internationally | John C. Radcliffe | **2019** |
| **33** | Infrastructure and Urban Planning Context for Achieving the Visions of Integrated Urban Water Management and Water Sensitive Urban Design: The Case of Melbourne | Casey Furlong, Meredith Dobbie, Peter Morison, Jago Dodson, Micah Pendergast | **2019** |
| **34** | Selection of the Best Water Supply Scenario for Urban Demand Based on the Risk Analysis in Decision-Making Model | Sabbaghian, RJ; Nejadhashemi, AP | **2019** |
| **35** | Integrated urban water management applied to adaptation to climate change | Paul Kirshen, Semra Aytur, Jory Hecht, Andrew Walker, David Burdick, Stephen Jones, Neil Fennessey, Renee Bourdeau, Lorilee Mather | **2018** |
| **36** | A Stakeholder-Science Based Approach Using the National Urban Water Innovation Network as a Test Bed for Understanding Urban Water Sustainability Challenges in the U.S. | J. Bolson, M. C. Sukop, M. Arabi, G. Pivo, A. Lanier | **2018** |
| **37** | Reasons for government inaction and its negative consequences: two case studies of failed water management initiatives in Alberta, Canada | Nicol, Lorraine A.; Nicol, Christopher J. | **2018** |
| **38** | Water quality monitoring in urban basins as support for water resources management: a case study from southern Brazil | Finkler, NR; Cocconi, J; Bortolin, TA; Mendes, LA; Schneider, VE | **2018** |
| **39** | Adapting Urban Water Systems to Manage Scarcity in the 21st Century: The Case of Los Angeles | Stephanie Pincetl, Erik Porse, Kathryn B. Mika, Elizaveta Litvak, Kimberly F. Manago, Terri S. Hogue, Thomas Gillespie, Diane E. Pataki & Mark Gold | **2018** |
| **40** | Green Infrastructure through Citizen Stormwater Management: Policy Instruments, Participation and Engagement | Lieberherr, E; Green, Olivia Odom | **2018** |
| **41** | Analysing the Role of Visions, Agency, and Niches in Historical Transitions in Watershed Management in the Lower Mississippi River | Tom Van der Voorn and Jaco Quist | **2018** |
| **42** | Aligning ancient and modern approaches to sustainable urban water management in China: Ningbo as a “Blue-Green City” in the “Sponge City” campaign | Y-T. Tang, F.K.S. Chan, E.C. O'Donnell, J. Griffiths, L. Lau, D.L. Higgitt, C.R. Thorne | **2018** |
| **43** | A participatory sustainability assessment for integrated watershed management in urban China | Brornbal, D; Niu, Y; Pizzol, L; Moriggi, A; Wang, JZ; Critto, A; Jiang, X; Liu, BB; Marcomini, A | **2018** |
| **44** | New Planning Activities in Vienna's Water-Management | Dokulil, MT; Donabaum, K; Teubner, K | **2018** |
| **45** | Green infrastructure for sustainable urban water management: Practices of five forerunner cities | Liu Li, Jensen Marina Bergen | **2018** |
| **46** | Strategies for developing transformative capacity in urban water management sectors: The case of Melbourne, Australia | Christoph Brodnik, Rebekah Brown | **2018** |
| **47** | Socio-environmental drought response in a mixed urban-agricultural setting: synthesizing biophysical and governance responses in the Platte River Watershed, Nebraska, USA | Zipper, SC; Smith, KH; Breyer, B (Breyer, Betsy); Qiu, JX; Kung, A; Herrmann, D | **2017** |
| **48** | Suitability assessment of the urban water management transition in the Indonesian context - A case study of Surabaya | Mar’atus Sholihah; Maria Anityasari; Diesta Iva Maftuhah | **2017** |
| **49** | Urban Waterfront Revivals of the Future | Swinal Samant & Robert Brears | **2017** |
| **50** | Towards water sensitive cities in Asia: an interdisciplinary journey | N. J. Barron; M. Kuller; T. Yasmin; A. C. Castonguay; V. Copa; E. Duncan-Horner; F. M. Gimelli; B. Jamali; J. S. Nielsen; K. Ng; ... Show more | 2017 |
| **51** | Examining water quality effects of riparian wetland loss and restoration scenarios in a southern ontario watershed | Yang, WH; Liu, YB; Ou, CP; Gabor, S | **2016** |
| **52** | Implementing integrated water management: illustrations from the Grand River watershed | Barbara Veale &Sandra Cooke | **2016** |
| **53** | Perspectives on policy framework for trans-boundary water quality management in China | Zhang, W; Zhen, GC; Tong, YD; Yang, L; Zhua, Y; Liu, GH; Wang, XJ; Li, Y | **2016** |
| **54** | Case Studies of the Sponge City Program in China | Li, XN; Li, JQ; Fang, X; Gong, YW; Wang, WL | **2016** |
| **55** | Modular Concept for Municipal Water Management in the Kharaa River Basin, Mongolia | Daniel Karthe, Sonja Heldt, Grit Rost, Jörg Londong, Jens Ilian, Jörn Heppeler, Jürgen Stäudel, Ganbaatar Khurelbaatar, Chris Sullivan, Manfred van Afferden, Buren Scharaw, Thomas Westerhoff, Steffen Dietze, Katja Sigel, Jürgen Hofmann, Vanessa Watson & Dietrich Borchardt | **2016** |
| **56** | Life cycle assessment of forecasting scenarios for urban water management: A first implementation of the WaLA model on Paris suburban area | Loubet, P; Roux, P; Guerin-Schneider, L; Bellon-Maurel, V | **2016** |
| **57** | Istanbul: the challenges of integrated water resources management in Europa’s megacity | Kees van Leeuwen & Rosa Sjerps | **2016** |
| **58** | A structure-efficiency based performance evaluation of the urban water cycle in northern China and its policy implications | Chu, JY; Wang, JH; Wang, C | **2015** |
| **59** | IWRM in a country under rapid transition: lessons learnt from the Kharaa River Basin, Mongolia | Karthe, D; Heldt, S; Houdret, A; Borchardt, D | **2015** |
| **60** | Stakeholder participation to watershed management: A case study from Beysehir Lake Basin | Yavuz, F; Baycan, T | **2015** |
| **61** | Sustainability transitions in the developing world: Exploring the potential for integrating Sustainable Urban Drainage Systems in Sub-Saharan Cities | Patience Mguni | **2015** |
| **62** | The water crisis and socio-ecological development profile of Rafsanjan Township, Iran | Mehryar, S; Sliuzas, R; Sharifi, A; van Maarseveen, | **2015** |
| **63** | Total water management and water sensitive cities | Dasith R. Gamage | **2014** |
| **64** | Urban-Water Harmony model to evaluate the urban water management | Ding, YF; Tang, DS; Wei, YH; Yin, S | **2014** |
| **65** | Liquid Assets V: The Water Tales of Hong Kong and Singapore: Divergent Approaches to Water Dependency | Su Liu; Jessica Williams | **2014** |
| **66** | The long road to improving the water quality of the Western Bug River (Ukraine) - A multi-scale analysis | Hagemann, N; Blumensaat, F; Wahren, FT; Trumper, J; Burmeister, C; Moynihan, R; Scheifhacken, N | **2014** |
| **67** | The Expanding Institutional Context for Water Resources Management: The Case of the Grand River Watershed | Ryan Plummer, Andrew Spiers, John Fitz Gibbon & Jack Imhof | **2013** |
| **68** | Water Resources Sustainability Indicator: Application of the Watershed Characteristics Approach | Peterson, HM; Nieber, JL; Kanivetsky, R; Shmagin, B | **2013** |
| **69** | Using the Storm Water Management Model to predict urban headwater stream hydrological response to climate and land cover change | Wu, JY; Thompson, JR; Kolka, RK; Franz, KJ; Stewart, TW | **2013** |
| **70** | Relationship between Water Withdrawals and Freshwater Ecosystem Water Scarcity Quantified at Multiple Scales for a Great Lakes Watershed | Mubako, ST; Ruddell, BL; Mayer, AS | **2013** |
| **71** | A framework for assessing effective urban water management: lessons from the Canadian Prairie | Jostein Kevinsen, Robert J. Patrick & Lalita A. Bharadwaj | **2013** |
| **72** | Action Research's Potential to Foster Institutional Change for Urban Water Management | Zikos, D; Thiel, A | **2013** |
| **73** | Diagnosing transformative change in urban water systems: Theories and frameworks | Briony C. Ferguson, Rebekah R. Brown, Ana Deletic | **2013** |
| **74** | Authority Structures and Service Reform in Multilevel Urban Governance: The Case of Wastewater Recycling in California and Australia | Sara Hughes | **2012** |
| **75** | Contiguous urban rivers should not be necessarily submitted to the same management plan: the case of Tietê and Pinheiros Rivers (São Paulo-Brazil) | Davi G. F. CunhaI; Doron GrullII; Murilo DamatoII; José R. C. BlumII; Sergio EigerIII; José E.I. LuttiIV; Pedro C. S. MancusoIII | **2011** |
| **76** | Transboundary Water Pollution Management: Lessons Learned from River Basin Management in China, Europe and the Netherlands | Xia Yu | **2011** |
| **77** | Total Water Management: The New Paradigm for Urban Water Resources Planning | Thomas P. O'Connor; Dan Rodrigo ; and Alek Cannan, | **2010** |
| **78** | Perspectives on small watershed management in China: the case of Biliu | Yong Geng, Bruce Mitchell, Fujita Tsuyoshi & Tadanobu Nakayama | **2010** |
| **79** | Watershed Governance Concept Backgrounder | Oliver M Brandes | **2006** |
| **80** | A sustainable framework for water resources management in an urban watershed: the case of Volos, Greece | N Mylopoulos & A Mentes | **2005** |
| **81** | Patterns of watershed urbanization and impacts on water quality | Carle, MV; Halpin, PN; Stow, CA | **2005** |
| **82** | Urban Water Resources Management in the Yellow River Basin: Perspectives of Sustainability | Xu, ZX | **2005** |
| **83** | Reexamining best management practices for improving water quality in urban watersheds | Pennington, SR; Kaplowitz, MD; Witter, SG | **2003** |
| **84** | Basic criteria for a sustainable water management at the U.S.-Mexico border: the case of Ambos Nogales | Luis Ernesto Cervera Gómez, Rodolfo Rubio Salas | **2003** |
| **85** | Forest cover, impervious-surface area, and the mitigation of stormwater impacts | Booth, DB; Hartley, D; Jackson, R | **2002** |
| **86** | Sustainable Watershed Management: An International Multi-Watershed Case Study | Walter Wagner, James Gawel, Hiroaki Furumai, Marcelo Pereira De Souza, Denilson Teixeira, Leonardo Rios, Shinichiro Ohgaki, Alexander J. B. Zehnder and Harold F. Hemond | **2002** |
| **87** | Dual urban and rural hydrograph signals in three small watersheds | Sheeder, SA; Ross, JD; Carlson, TN | **2002** |
| **89** | Integrated watershed approach for combating drought in a semi-arid region of India: the case of Jhabua watershed | A.K. Singh; T.I. Eldho; D. Prinz | **2002** |
| **90** | Towards more effective integrated watershed management in Australia: Results of a national survey, and implications for urban catchment management | Bruce Hooper | **2002** |
| **91** | Techniques for detecting hydrologic change in high resource streams | Hartley, DM; Funke, DE | **2001** |
| **92** | The Rouge Project: Implementing a General Storm Water Permit as Part of a Watershed Approach to Wet Weather Pollution Management | James E. Murray and Kelly A. Cave, Dale S. Bryson, Jack D. Bails | **1999** |
| **93** | The Joshua's Creek urban water resources management experience paradigm for sustainability or slave to expediency? | Bishop, R | **1999** |
| **94** | Substate institutional innovation for managing lakes and watersheds - a Wisconsin case-study | Nakamura, L; Born, SM | **1993** |
