# Supplementary material for: Natural dynamics and watershed approach incorporation in urban water management: A scoping review
Source: PLoS One. 2024 Aug 30;19(8):e0309239. doi: 10.1371/journal.pone.0309239 (PMC11364295; doi:10.1371/journal.pone.0309239)
Supplement: Attachment — Submitted filename: ReviewerComments_Apr2024.docx [file pone.0309239.s004.docx]

This paper applies a well described systematic methodology to reviewing existing literature focused on the implementation of watershed management approaches in urban settings. I believe the methodology is generally sound and well described. However, I have some concern that this methodology may have limited the number of relevant studies included in the review (and this is acknowledged by the authors). More importantly, as there are a number of existing publications, including review papers, that focus on watershed management approaches, I think some additional thought and discussion of what makes this review different would be valuable to readers. The answer to the question “What is this paper offering that others have not?” should be immediately identifiable. In addition, I believe some further editing work would improve readability and make the main points of this review more impactful.

1. General editing, while I have provided a few examples and suggested revisions here, I would suggest a thorough editing of the entire paper to ensure a consistent writing style, consistent use of tense, improve succinctness and reduce redundancy, and improve clarity of sentences and paragraphs. Each paragraph should have a primary idea/message that it communicates to readers. Below are some examples:
   a. Lines 50-51 (particularly important example b/c lead paragraph/sentence): “because of their need to obtain water to supply their population, their commercial and productive activities, and their industry, as well as facing the scarcity of this resource” -> “because of the conflicting demands on and availability of this resource. While urban centers have high needs due to their higher population densities as well as commercial and industrial activities, they often have higher scarcity of this resource due to {maybe add reason here}”.
   b. From abstract and lines 23-25: “Although the importance of a watershed approach in land management is acknowledged, when studies focus on water management, they generally study cases in rural areas or single case-studies of cities or countries”	-> “Although the importance of a watershed approach in land management is generally acknowledged, studies on this topic have typically focused on water management in rural areas or single case-studies of cities/countries.”
   c. Lines 21-22: “Some cities, however, have intended to incorporate a watershed approach in water management to seek more sustainable solutions.” -> “Some cities have incorporated a watershed approach to water management in seeking more sustainable solutions.”
   d. Lines 70 -71: “This risks urban water being contaminated by microbiological agents and organic and inorganic compounds…” -> “The risks include urban water being …”
   e. New paragraph at line 92 with focus on transport/regional effects of urbanization? There are a few sentences on the effects of urbanization sprinkled through the introduction – maybe consolidate for more impact?
   f. Lines 391-393 seem a bit contradictory – states that the primary issue is water quantity issues, but then water quality is listed as a motivation behind that. This paragraph could use clarification. I think the overall structure you are going for is a paragraph on each of the items in Table 2? If so, perhaps try to stick with that and focus as tightly as you can within each paragraph.
   g. Line 423: “…in Chinese’s cases, …” – should be either “…in China’s cases,…” or “…in Chinese cases,…”
   h. Line 486-488: “…which triggered that Oregon’s Department of Environmental Quality (DEQ) ordered the reduction of water pollution to the city of Portland ” ->”…which triggered Oregon’s Department of Environmental Quality (DEQ) to order the reduction of water pollution in(?) the city of Portland.”
   i. Lines 499-501: “These actions included converting impermeable gray spaces existent in cities, increase infiltration for water availability and floods control, using urban design modifications.” -> Unless I’ve misunderstood, the use of commas here is incorrect -> “These actions included conversion of impermeable gray spaces in cities to increase infiltration for water availability and flood control using urban design modifications.”
   j. Lines 501-503: “Also, this type of actions included the implementation of new infrastructure in cities incorporating the water cycle as a priority since project design, requiring a strong collaboration of land use planners with water resource managers.” -> “Actions also included implementation of new infrastructure in cities that considered the water cycle as a priority from project design stages, requiring a strong collaboration between land use planners and water resource managers.”
   k. Review uses of clauses like ‘Thus’, ‘Furthermore’, ‘In order’, ‘With regard to’, ‘Moreover’ -> these are often not necessary and in some cases not used correctly, taking away from the main point of sentences.
2. Tables/Methodology
   a. Table 1: add state to other US cities
   b. Table 2, 3, 4, and 5: I’m struggling to understand the % number here – perhaps explicitly state the denominator. It is also unclear whether number of cases indicates number of cities/urban centers where this was the driver, or number of studies more generally? Can multiple drivers have been identified for a single urban center?
   c. In the text (primarily Methodology section), does ‘case’ refer to a single city/urban center, or a single study, or are these the same?
3. Discussion
   a. The authors extensively discuss the lack of studies in Latin America. But there seems like other gaps as well (e.g. efforts in Canada, Japan). Perhaps this could be explored a bit more – why did the search criteria leave these out and what are the impacts? Was this a methodology limitation?
   b. The methodology is described from the standpoint of the drivers, actions, challenges, and results. However, the discussion is not clearly framed in the same way so I find it a bit challenging to tie together the results and discussion directly.
   c. I think there’s something interesting here related to the lack of evidence on results of implementation, and lack of metrics, that could be brought up in the discussion as well.
4. Conclusions
   a. Highlight here again what this paper offers that others haven’t and then the main take-aways, as supported by the review process implemented here. This should not just be a summary of the paper, it should be the key findings and perhaps what can we build on, etc.
